# The Functional Architecture of the Brain Underlies Strategic Deception in Impression Management

**DOI:** 10.3389/fnhum.2017.00513

**Published:** 2017-11-02

**Authors:** Qiang Luo, Yina Ma, Meghana A. Bhatt, P. Read Montague, Jianfeng Feng

**Affiliations:** ^1^School of Life Sciences, Fudan University, Shanghai, China; ^2^Institute of Science and Technology of Brain-Inspired Intelligence, Fudan University, Shanghai, China; ^3^State Key Laboratory of Cognitive Neuroscience and Learning, International Data Group, McGovern Institute for Brain Research, Beijing Normal University, Beijing, China; ^4^Beijing Key Laboratory of Brain Imaging and Connectomics, Beijing Normal University, Beijing, China; ^5^Virginia Tech Carilion Research Institute, Roanoke, VA, United States; ^6^Department of Neuroscience, Baylor College of Medicine, Houston, TX, United States; ^7^Wellcome Trust Centre for Neuroimaging, University College London, London, United Kingdom; ^8^Shanghai Center for Mathematical Sciences, Shanghai, China; ^9^Department of Computer Science, University of Warwick, Coventry, United Kingdom; ^10^Collaborative Innovation Center for Brain Science, Fudan University, Shanghai, China

**Keywords:** impression management, economic games, signal-dependent noise, directional connectivity, support vector machine

## Abstract

Impression management, as one of the most essential skills of social function, impacts one's survival and success in human societies. However, the neural architecture underpinning this social skill remains poorly understood. By employing a two-person bargaining game, we exposed three strategies involving distinct cognitive processes for social impression management with different levels of strategic deception. We utilized a novel adaptation of Granger causality accounting for signal-dependent noise (SDN), which captured the directional connectivity underlying the impression management during the bargaining game. We found that the sophisticated strategists engaged stronger directional connectivity from both dorsal anterior cingulate cortex and retrosplenial cortex to rostral prefrontal cortex, and the strengths of these directional influences were associated with higher level of deception during the game. Using the directional connectivity as a neural signature, we identified the strategic deception with 80% accuracy by a machine-learning classifier. These results suggest that different social strategies are supported by distinct patterns of directional connectivity among key brain regions for social cognition.

## Introduction

How we are viewed by other people has a significant impact on our daily social interactions (Lyle and Smith, 2014). A positive social image affects one's survival and success in larger-scale societies, for example, it helps individuals to achieve a successful career, develop satisfying relationships and gain greater social support (Lyle and Smith, 2014). Individuals take different actions to manage their social images in other's mind, such as showing trust on others, punishing those who take free ride, engaging in costly cooperation, mimicking others' behavior, or employing strategic deception (Panchanathan and Boyd, 2004; Bhatt et al., 2010; Phan et al., 2010; Jordan et al., 2016). However, the neural architecture underlying these different strategies to manage impression remains poorly understood.

To build up and manipulate impression require us to model the beliefs and desires of other people (Frith and Frith, 2005; Amodio and Frith, 2006; Sanfey, 2007) and to send signals that could manipulate others' perceptions of ourselves (Tennie et al., 2010). This kind of modeling and impression management sometimes require sophisticated strategies. Recently, cognitive probes paired with function magnetic resonance imaging (MRI) have begun to reveal neural underpinnings of impression management and to decompose the component computation or different strategies involved in such sophisticated exchanges (King-Casas et al., 2005; Behrens et al., 2009). In line of recent works on the neural responses engendered during social interactions (Keysers and Gazzola, 2007; Bara et al., 2011; Manera et al., 2011), several brain areas have been identified as parts of a possible “theory of mind” network (Carrington and Bailey, 2009). For example, positive social images were associated with stronger neural activation in the reward system, including ventral striatum and orbitofrontal cortext (Delgado et al., 2005; Phan et al., 2010; Fouragnan et al., 2013). Four such response profiles were highlighted in a recent study by Bhatt et al. (2010). Using a simple model for categorizing the interpersonal exchange during a two-party bargaining game, subjects in our experiment fell into three distinct behavioral groups that depended on their strategic sophistication during the bargaining: (1) incrementalists, (2) conservatives, and (3) strategists or strategic deceivers. The “incrementalists” employed a simple strategy of anchoring their social signals to the truth, and showed stronger activation in the dorsal anterior cingulate cortex (dACC). Mimicking a more benign behavioral type, individuals with high level of strategic deception employed a sophisticated strategy to build up a positive impression. These strategists showed stronger activation in the right dorsolateral prefrontal cortex (DLPFC), rostral prefrontal cortex (rPFC or BA10) and retrosplenial cortex (RSC), but less activation at the middle paracingulate cortex (MPC). The “conservatives” chose not to send any useful information at all.

Most literatures have focused on identifying differentially activated brain regions by distinct social strategies, however, little is known about how the responses in these brain regions might interact. Brain functional integration can be investigated by the statistical models of the information flows traveling among brain regions (Friston, 2002). It has been recently shown that complex social processes are supported by different brain networks (Park and Friston, 2013), such as motives behind human altruism (Hein et al., 2016). Currently, much of our understanding of the directional connectivity among brain regions comes from the mathematical modeling of brain networks (Friston, 2002). For example, the classical Granger causality analysis (GCA) estimates the directional connectivity via an autoregressive model (Ding et al., 2006), and the dynamic causal modeling (DCM) approximates the dynamic interaction between brain areas with a bilinear model (Friston et al., 2003). However, one possible over-simplification in some scenarios is that the noise process in neural signal has been assumed to follow a time invariant model. As the spike train of a neuron is typically close to Poisson processes in their timing, the variance thus increases linearly with the signal (Gerstein and Mandelbrot, 1964). In addition, such signal-dependent noise (SDN) has been demonstrated to be functionally important, for example, as an optimal control strategy for motor planning (Harris and Wolpert, 1998). However, in presence of the SDN in the blood-oxygen-level dependent (BOLD) signal recorded from the fMRI experiments (Luo et al., 2013), neither the classical GCA nor the DCM is applicable, as both models assuming a constant-level of variance for the brain signal. Therefore, we have proposed an adaptation of the classical GCA to model the SDN, and be thus sensitive to the variance-affected interactions that other methods would miss (Luo et al., 2011, 2013). Applying this model to the fMRI data of the two-party bargaining game, we uncovered the information flows traveling among key brain regions mediating the impression management, and accurately identified the strategic deception during social interaction using the estimated directional connectivity as a neural signature.

## Materials and methods

### Participants

A total of 76 healthy participants (36 females; Mean ± SD age = 29.6 ± 7.6; Mean ± SD *IQ* = 117.2 ± 19.4; Mean ± SD social economic status = 42.8 ± 3.5) participated in the accordance with a protocol approved by the Baylor College of Medicine Institutional Review Board. Informed consent was obtained from all participants, and all procedures were in accord with the ethical standards set out in the Declaration of Helsinki. We refer to our previous studies (Bhatt et al., 2010) for more details of the participants.

### The no-feedback bargaining game

**Figure 1:**
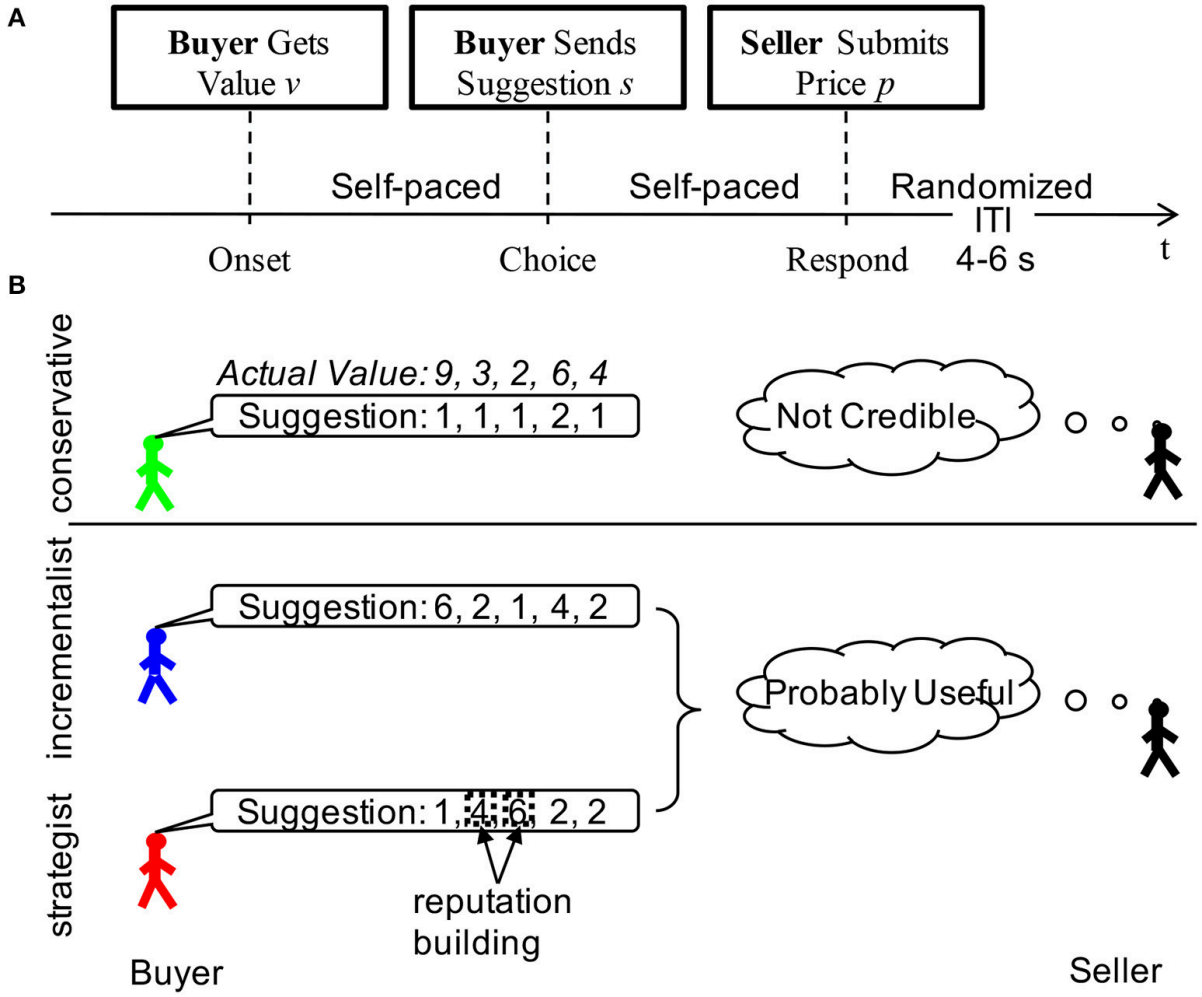
Two-person bargaining game. **(A)** Task design; **(B)** Reputation formation in different behavioral groups. Although, there was no feedback in the task, sellers saw the stream of suggestions sent by the buyers. They could make inferences about the buyer based on these streams. In the example above we demonstrated the suggestions that might be sent out by each of the three behavioral types for a fixed sequence of underlying values. Noticed that a seller could easily infer from the low variance of the conservatives suggestions that they were unlikely to convey any useful information. On the other hand, the incrementalist's suggestions conveyed a great deal of information about the values and a seller could use these suggestions to his benefit. The strategist took advantage of the fact that sellers were more likely to trust and use suggestions when the stream of suggestions had high variance. They could use the trials when the actual values were low in this example when they were 3 and 2 to build their reputation by sending higher suggestions, mimicking an incrementalist profile. When the values were high, they could send relatively low suggestions and take advantage of the credibility they had built during the low-value trials. Noticed that from the seller's perspective, the incrementalists and strategists were essentially indistinguishable, sending the same mixture of suggestions, however the relationship of these suggestions to the underlying values were completely different.

In this game, two players, a buyer and a seller, played 60 rounds of a bargaining task (Figure 1A). The duration of each trial was self-paced depending on how long it took for the participant to respond. The inter-trial-interval was randomized according to a uniform distribution from 4 to 6 s. The scan continued till the participants finished all 60 rounds of bargaining trials. For each bargaining trial, the “buyer” was first given the private value *v* of a hypothetical object by a computer. The buyer was then asked to “suggest a price” to the seller (values and prices were integers, 1–10). After the seller received the suggestion, she/he was asked to offer a price *p*. If the offered price was less than the private value of the object, the trade executed, and the seller and the buyers received values of *p* and *v*-*p*, respectively; otherwise, the trade failed and no one got any value. No feedback about whether the trade executed was provided to either the seller or the buyer. However, despite the lack of feedback, sellers often did form inferences about buyer credibility based on the stream of suggestions sent and many buyers were aware of this fact, prompting them to attend to maintaining the appearance of credibility (Figure 1B). As shown in Figure 1B, the sellers got all their information about the value of the item from the buyers. When the seller saw a sequence of varying prices suggested by the buyers during the game, they might think that the buyers were suggesting prices according to the true values of the item. Indeed, some buyers (the incrementalists) did anchor their suggesting prices to the true values so as to share the rewards with the sellers. However, the strategist group also sent out a sequence of varying prices, only that they suggested high prices for items with low values but low prices for items with high values. It was difficult for the sellers to tell the difference between these two strategies from the observation of a sequence of varying prices without any feedback. Of course, it was easy for the sellers to identify the conservatives, as they sent out constantly low prices all the time.

To model the bargaining strategy during the game, the buyers' suggestions were regressed on the real values of the items by a linear model after their behavior stabilized in the game. From these linear regressions, two behavioral characteristics were extracted, i.e., the slope and the R^2^ of the regression, and were used to identify different behavioral groups in buyers by a clustering algorithm. The slope of the linear model was used as a parameter of information relevance representing the bargaining strategy, and was referred as a buyer's “information revelation” coefficient (IR). We used the *k*-means in a two-dimensional feature space, consisting of the IR and the R^2^, where *k* = 3.

Both players received their aggregate earnings over 60 trials at a predetermined exchange rate at the end of the experiment. This is a hyper-scanning experiment, i.e., each player was in an fMRI scanner for the entire session while they were playing the designed bargain game in real-time. The brain activation patterns had been reported previously for both the buyers (Bhatt et al. 2010) and the sellers (Bhatt et al., 2012). Here in this paper, we focused on the neural network architecture in buyers, and the effects of the social interaction on the modulation of directional connectivity would be carried out in a separate study. As reported previously (Bhatt et al., 2010), no significant correlation had been identified between the final rewards collected by the buyers and their characterizations, including age, IQ, socioeconomic status, and gender. In our analysis, we did not find any correlation between these characterizations and the estimated directional connectivity.

### Image acquisition and preprocessing

The fMRI data were collected using 3-T Siemens scanners. High-resolution T1-weighted scans were acquired using a magnetization prepared rapid gradient echo sequence. Whole-brain echo planar images were acquired with BOLD contrast and a repetition time of 2,000 ms (echo time, 25 ms). Thirty-seven 4-mm slices were acquired 30 degrees off the anteroposterior commissural line, yielding 3.4 × 3.4 × 4.0 mm voxels. The fMRI data were preprocessed in our previous study (Bhatt et al., 2010) using the SPM (http://www.fil.ion.ucl.ac.uk/spm) with standard procedures, such as slice-timing correction, motion correction, coregistration, normalization to the Montreal Neurological Institute template, and high-pass filtered (128 s).

### fMRI time-series

Time series data were extracted and averaged over voxels in each of the regions of interest (ROIs), which were defined by the activation analysis in our previous study (Bhatt et al., 2010), including rPFC (−32, 48, 20), DLPFC (36, 28, 36), RSC (8, −56, 8), dACC (−4, 20, 32), and MPC (0, 4, 56). Brain activities in these regions were associated with different strategies for impression management in the two-person bargaining game. Particularly, compared with conservatives and incrementalists, strategists showed stronger activity in the rPFC and DLPFC, revealed by first-level boxcar regressors over the entire trial (onset to decision), and stronger RSC activity at the moment of deception. Moreover, stronger activations in the dACC and MPC were found in the incrementalists, relative to conservatives and strategists. Here, in this paper, we also estimated the brain activity of each region by comparing the signal during choice making with the signal at trial onset, and calculated the median of such ratios among the last 30 trials. The bargaining behavior wasn't stable at earlier trials, thus only the second half of the trials were used for the analysis, i.e., only the last 30 trials were used for each subject. The group difference in brain activity can be tested by one-way analysis of variance (ANOVA).

In order to carry out the analysis of directional connectivity, we controlled for the event-induced dynamic, since this dynamic might constitute a common driver of brain activities in all these brain regions. Here, we convolved the event train with a hemodynamic response function (HRF), particularly, the fourth order Fourier HRF established by SPM8, and then regressed out this event signal from all brain regions. Next, BOLD signals were detrended. As recommended by Wen et al. (2012), we did not regress out the head motion from the time series to prevent from over-preprocessing. In line with the literature (Johnstone et al., 2006; Schreiber and Krekelberg, 2010; Wen et al., 2012), regressing out the head motion parameters would lead to spurious activation effects, especially when these parameters are correlated with experimental design (event train). In our case, we first convolved the event train with the HRF (4-th Fourier series) and then down-sampled to the same sampling rate as the motion parameters; and second, we calculated the correlation between the motion parameters and the experiment design. The percentages of significant correlation (*p* < 0.05) between motion parameters (translation and rotation) and the event trains were high for three event trains: trail onset (66.5%), choice making (68.4%), key pressing (90.1%). Therefore, regression out the motion parameters would compromise the experiment design in our case and render our analysis to be insensitive. However, we did compare the head motion parameters for both displacement (*p* = 0.55) and rotation (*p* = 0.82) among three behavioral groups, and no significant group difference were observed. Also, we had calculated the behavioral correlation between the motion parameters and the information revelation (IR) both in all subjects and in each group, and no correlation reached any significant level. Therefore, in our case, the identification of group comparison and behavioral correlation are unlikely to be confounded by the head motion. However, we did compare the head motion parameters for both displacement (*p* = 0.55) and rotation (*p* = 0.82) among three behavioral groups, and no significant group difference were observed. Also, we had calculated the behavioral correlation between the motion parameters and the information revelation (IR) both in all subjects and in each group, and no correlation reached any significant level. Therefore, in our case, the identification of group comparison and behavioral correlation are unlikely to be confounded by the head motion. Inter-trial scans were then excluded, limiting our attention to the time-series data for choice-making trials of the buyers. Each observation consisted of the time-series from one trial, beginning at the trial onset, and ending when the buyer made the choice. For each trial, the time series were also demeaned. Here, we are going to use the Granger causality model with SDN for the non-stationary time series (Luo et al., 2011), that has been demonstrated to be a promising tool for causal inference on both simulated and experimentally collected BOLD signals (Luo et al., 2013).

### Detection of signal-dependent noise

To detect if the SDN existed in the BOLD signals, we first estimated a standard autoregressive (AR) model to our data and examined the correlation between the residual process of this analysis and the squared signals of the time-series. If the AR model fits the data, the variance level of the residual process would be Gaussian white noise with no temporal correlation with the signal; Otherwise, if a significant correlation has been observed between the residual process and the strength of the signal with one time lag, the residual process is not a Gaussian white noise process and the precondition of the classic AR model is not satisfied. Mathematically, for the BOLD signal observed at each brain region, the strength of the noise (or, residual) process at each time step was measured by the squared residual process, ${û}_{t}^{2}$, established by the autoregressive model. The AR can be formulated as $X_{t}=\overset{p}{\underset{i=1}{\sum }}A_{i}X_{t-i}+u_{t}$, where *A*_*i*_ is the model coefficient and *u*_*t*_ is the Gaussian white noise process with a constant variance. Using the least-square estimation, we can estimate the model coefficient as Â_*i*_, and then the residual process is given by ${û}_{t}=X_{t}-\overset{p}{\underset{i=1}{\sum }}{Â}_{i}X_{t-i}$. The strength of the lagged signal is measured using the squared signal with one time lag, $X_{t-1}^{2}$. Outliers of the data ($X_{t-1}^{2}$, ${û}_{t}^{2}$) were filtered out using a criterion of 3-sigma. By pooling the data for all five regions together, the variance level of the noise process, ${û}_{t}^{2}$, could be plotted against the strength of the BOLD signal with one time lag, $X_{t-1}^{2}$.

To avoid the possibility that the observed correlation was a model-specific result, we used two alternative models for the BOLD signal to assess the SDN. The residual processes were established, respectively by ${û}_{t}=X_{t}-{Â}_{i}X_{t-1}-{\hat{B}}_{1}X_{t-1}^{2}$ and û_*t*_ = *X*_*t*_−Fourier6(*X*_*t*−1_), where Fourier6(·) was the Fourier series in the 6th order.

### Granger-causality with SDN

To deal with the SDN observed in BOLD signal, we proposed a new algorithm (Luo et al., 2011) and successfully applied to fMRI data analysis to identify different directional networks modulated by biased attentions in our previous study (Luo et al., 2013). The proposed algorithm was based on a simple idea of prediction as the classical Granger causal modeling (Granger, 1969). If including the history information of time series *Y* improves the prediction accuracy of time series *X*, then changes in *Y* cause changes in *X*. Mathematically, suppose we have time series data from two ROIs, *X* and *Y*. We want to infer whether *Y* causally influences *X*. Consider the following two models for *X*:

$$X_{t}=\sum_{i=1}^{p}A_{xx,i}X_{t-i}+r_{xx,t},\;r_{xx,t}=H_{xx,t}^{1/2}u_{xx,t},$$

$$H_{xx,t}={C^{\prime }}_{\text{​}xx}C_{xx}+\sum_{i\;=\;1}^{q}{B^{\prime }}_{\text{​}xx,j}X_{t-j}{X^{\prime }}_{t-j}B_{xx,j},$$

and,

$$\begin{array}{l} X_{t}=\sum_{i\;=\;1}^{p}A_{xy,i}X_{t-i}+\sum_{i\;=\;1}^{p}D_{xy,i}Y_{t-i}+r_{xy,t},\\ r_{xy,t}=H_{xy,t}^{1/2}u_{xy,t}, \end{array}$$

$$H_{xy,t}={C^{\prime }}_{\text{​}xy}C_{xy}+\sum_{i\;=\;1}^{q}{B^{\prime }}_{\text{​}xy,j}{\left[{X^{\prime }}_{t-j},{Y^{\prime }}_{t-j}\right]}^{\prime }[{X^{\prime }}_{t-j},{Y^{\prime }}_{t-j}]\;B_{xy,j},$$

where *p* and *q* are the model orders, *A*_*xx, i*_*, A*_*xy, i*_*, D*_*xy, i*_*, i* = *1, …, p, B*_*xx, j*_*, B*_*xy, j*_*, j* = *1, …, q* and *C*_*xx*_, *C*_*xy*_, are all coefficient matrices and *u*_*xx*_*, u*_*xy*_ are Gaussian white noise processes. Clearly, if the coefficients *B*_*xx, j*_ and *B*_*xy, j*_ are all zeros, these models are deduced to the classical autoregressive (AR) models, since the signal-dependent variances of the noise processes, *r_xx,t_* and *r_xy,t_*, become constants in this case. Here the *H* matrices describe the predicted noise for each model. Since the above models for *H* are similar to the BEKK (named after Baba, Engle, Kraft, and Kroner) model for time-varying volatility (Baba et al., 1991), we refer to these models as AR-BEKK models. The first model assumes no causal influence from *Y* to *X*, while the second allows *Y* to affect both the signal and the variance of *X*. Notice that in these models the errors are modeled in more detail than in a classical Granger setup, so the level of unexplained error for each of these models are represented by *C*_*xx*_, and *C*_*xy*_, respectively. Thus, we can define a causality statistic as below, the larger the statistic, and the stronger the directional connectivity.

$$F_{Y\to X}=\log\frac{\text{trace}[{C^{\prime }}_{\text{​}xx}C_{xx}]}{\text{trace}[{C^{\prime }}_{\text{​}xy}C_{xy}]}$$

Given the TR was 2 s, we used *p* = *q* = 1 in this analysis following the literature (Wen et al., 2012; Luo et al., 2013; Ding et al., 2015). For more details regarding to the model of the Granger causality with SDN and its validation in both simulated and experimentally collected BOLD signals, we refer to our previous studies (Luo et al., 2011, 2013). A Matlab toolbox of this algorithm is also available at http://www.dcs.warwick.ac.uk/~feng/causality.html.

### Group difference and behavioral correlation

To find the most informative brain interaction from all directional connectivity estimated, we first tested whether the directional connections among the 5 ROIs (i.e., 20 directions) were significantly >0 by one sample *t*-test collapsing different strategy groups, and found that all the 20 directions were significantly connected. Next, we compared the directional connectivity between strategy groups and assessed the linear association between the directional connectivity and the bargaining strategic indices (e.g., the information revelation and the R^2^). Since the time-series data with SDN usually have high-level noise, the model might fail to give reliable estimation due to limited data points. In this analysis, we excluded those estimated causalities outside a range of ±2.7 standard deviations (approximately 99.3% coverage of a normally distributed data) from the mean causality at each direction from each strategic group. The average number of subjects excluded from each group at each direction was 1.5. Therefore, to identify group differences and behavioral associations the significance level was corrected for multiple comparisons among all 20 directions (Bonferroni correction, i.e., *p* < 0.05/20).

### Demonstration of the extra information provided by the brain circuit in addition to the brain activation

To demonstrate that the directional influences among brain regions provide extra information than the brain activations for us to understand the underlying neural circuits of strategic deception during the game, we built classification models with different input features and compared their performances in terms of classification accuracy for identifying strategic deception. Considering the potential interactions between the ROI's, we employed multivariate classifier instead of the linear models. Particularly, we used the Support Vector Machine (SVM) (Chih-Chung and Chih-Jen, 2011) implemented by LIBSVM, which can be downloaded at the following website http://www.csie.ntu.edu.tw/~cjlin/libsvm/. Using brain activities or directional connectivity as input features, we built three SVM's to identify the strategic deception from other behavioral types. The first model took input of five brain activations, the second model used the twenty directional connectivity as inputting features, and the third model combined the features in the first and the second models. The brain activity was estimated by comparing the signal during choice making with the signal at trial onset, and calculated the median of such ratios among the last 30 trials. The directional connectivity was estimated by the proposed GCSDN approach. In SVM, the linear kernel was used to reduce the model complexity and thereby to reduce the possibility of overfitting. The regularization parameter in the model was set to be 10 to get the best classification result by the first SVM model, and the other two models used the same set of parameters to ensure a fair comparison. We used leave-one-out procedure to prevent the problem of over-fitting, i.e., the model was trained in 75 subjects, but applied to the one subject left. The averaged accuracy of 76 leave-one-out experiments was used to measure the performance of the classifier. To further assess specificity and sensitivity of different classifiers, we plotted the receiver operating characteristic (ROC) curve for each classifier, and compared the area under curve (AUC) (Fawcett, 2006) among different classifiers. As the AUC has been considered to be too conservative to assess the improvement in classification by including new markers, we employed two new measures to evaluate the improvement in classification by adding directional connectivity to brain activation, the net reclassification improvement (NRI) and the integrated discriminative improvement (IDI) (Pencina et al., 2008). The NRI quantifies the correct movement in categories by including new features, i.e., reclassifying cases into case group and controls into control group; The IDI focuses on differences between average sensitivity and specificity for models before and after including extra new features. A significant improvement is identified when the statistical tests give a significant (<0.05) *p*-value (Pencina et al., 2008).

## Results

### Behavioral groups

By k-means in a two-dimensional feature space, consisting of the IR and the R2 (Bhatt et al., 2010), the buyers fell into three distinct behavioral groups depending on their strategic sophistication during the bargaining: (1) conservatives (*n* = 28), (2) incrementalists (*n* = 32), and (3) strategists (*n* = 16). The “conservatives”, characterized by IRs close to zero (Mean ± SD = 0.13 ± 0.23) and intermediate or low fit (Mean ± SD = 0.24 ± 0.18), chose not to send useful information at all. The “incrementalists,” who generally showed relatively high IRs (Mean ± SD = 0.57 ± 0.18) and high fit (Mean ± SD = 0.80 ± 0.15), employed a simple strategy of anchoring their social signals to the truth. The “strategists”, exhibiting a negative IR (Mean ± SD = −0.68 ± 0.21) and high fit (Mean ± SD = 0.54 ± 0.17), adopted a sophisticated, forward-looking strategy aiming at projecting an impression of trustworthiness first and next exploiting the trust built up in their partner (Figure 1B).

### fMRI data exhibits signal-dependent noise

**Figure 2:**
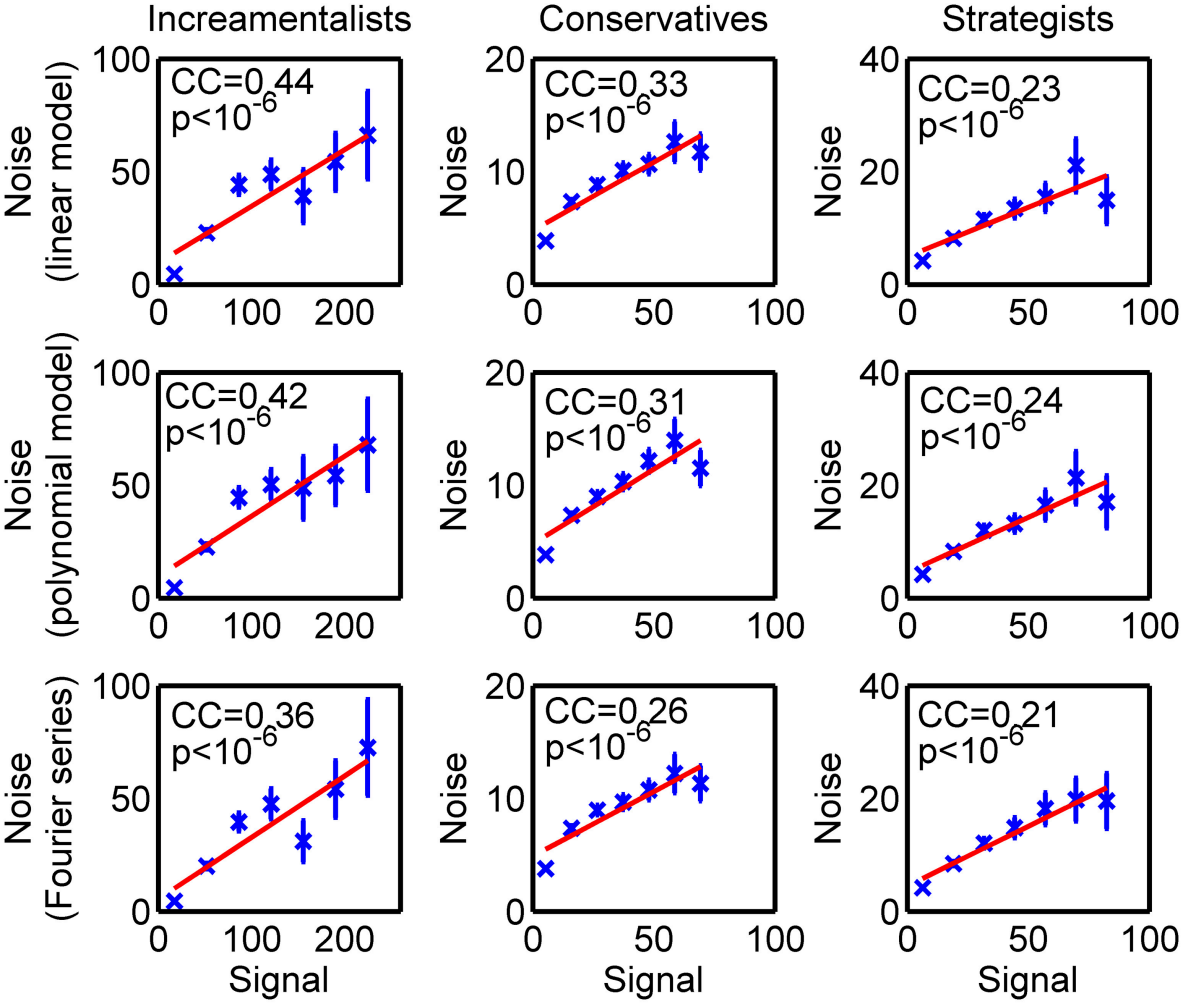
Signal-dependent noise. The strength of the signal at time *t*-1 was measured by the squared signal $X_{t-1}^{2}$, while the noise level of the residual process was given by the squared residual at time *t (*${û}_{t}^{2}$) by different models (presented as the title of each subplot) of the BOLD time series. The strengths of the signals were binned into 7 bins. For each bin, the corresponding mean value (crosses) and the corresponding standard error (error bar) of the noise levels in each bin were established. The lines are the linear fits of the mean values of the noise levels to the centers of bins of the strengths of the signals. The correlation coefficients (CC) between the noise level and the strength of the signal as well as the corresponding *p*-values (p) are also reported.

As demonstrated earlier that the BOLD signal in fMRI experiment can have the SDN (Luo et al., 2011, 2013), we tested if the BOLD signals of the brain ROI in the current study have such SDN. For the AR model used by the classical Granger causality, the significances of the correlation between model residual and signal were all smaller than 10^−6^ (Figure 2), which suggested that those models assuming a constant noise level in the BOLD signal (e.g., AR model) were not applicable to the current data. We increased the complexity of the model to see if the observed correlation was due to a miss specifying of the model for the BOLD signal. Neither including a second order term, nor using a highly nonlinear model (i.e., 6th order Fourier series) decreased the correlation (Figure 2). This result suggested that the observed SDN might be a nature of the BOLD signal.

### Group difference in directional connectivity between brain regions

**Figure 3:**
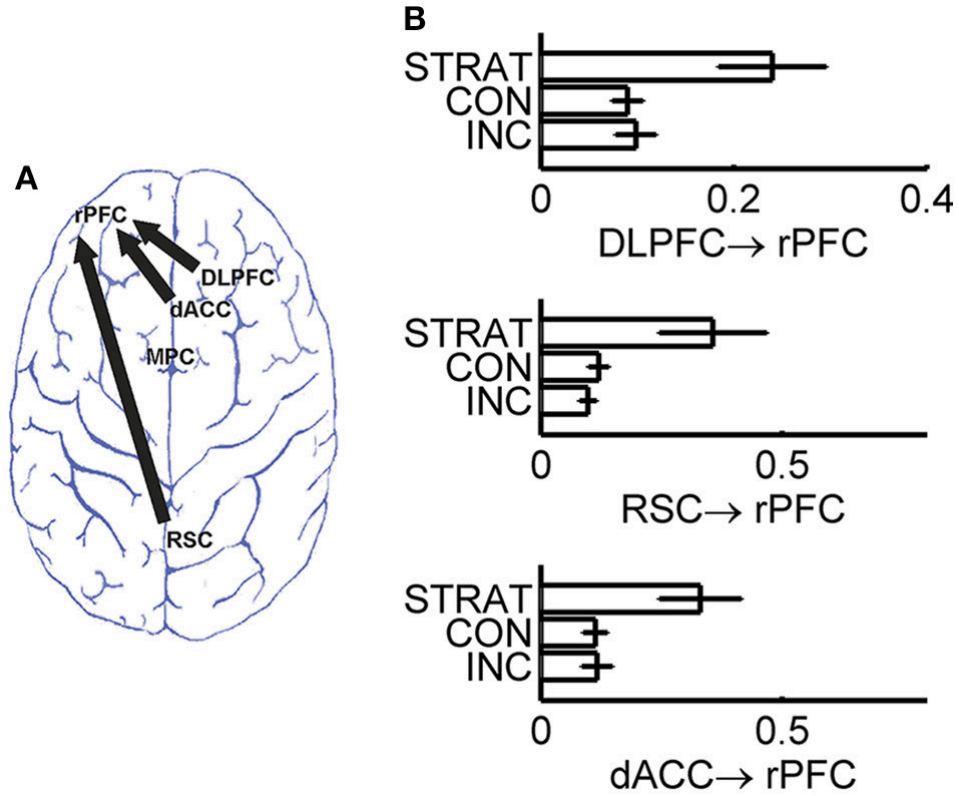
Directional connectivity with significant group difference after Bonferroni correction. **(A)** Brain map for three directional connectivity; **(B)** Group comparison of the strength of directional connectivity. INC, incrementalists; CON, conservatives; STRAT, strategists.

As information flows among brain regions can be estimated by directional connectivity between ROI's, we assessed the directional connectivity between each pair of these 5 brain regions underlying impression management by employing the Granger causality with SDN. To identify information flows responsible for strategic deception, we carried out one-way ANOVA with group (incrementalists, conservatives, or strategists) as a between-subject factor to reveal whether the directional interactions among these brain regions were significantly different among different strategic groups. We found that the directional connectivity from RSC to rPFC, from DLPFC to rPFC and from dACC to rPFC were significantly modulated by strategic groups (*p* < 0.0025, after Bonferroni correction for 20 directional connectivity). *Post-hoc* analyses confirmed that the identified directional connectivity were significantly stronger in the strategists than incrementalists and conservatives (Table 1 and Figure 3).

**Table 1:** Comparison of directional connectivity across three behavioral groups.

**Directional connectivity**	**INC**	**CON**	**STRAT**	***p*-value**	**Directional connectivity**	**INC**	**CON**	**STRAT**	***p*-value**
RSC—>rPFC	0.10	0.12	**0.36**	**0.0009**	rPFC—>RSC	0.11	0.14	0.18	0.3727
RSC—>MPC	0.17	0.07	0.10	0.0224	MPC—>RSC	0.10	0.10	0.11	0.9251
RSC—>rDLPFC	0.07	0.13	0.12	0.1086	rDLPFC—>RSC	0.10	0.09	0.13	0.5740
rDLPFC—>rPFC	0.10	0.09	**0.24**	**0.0016**	rPFC—>rDLPFC	0.08	0.10	0.07	0.7623
rDLPFC—>MPC	0.11	0.06	0.11	0.1820	MPC—>rDLPFC	0.15	0.13	0.16	0.8252
dACC—>rPFC	0.12	0.11	**0.33**	**0.0022**	rPFC—>dACC	0.05	0.17	0.07	0.0044
dACC—>MPC	0.16	0.07	0.22	0.0079	MPC—>dACC	0.11	0.15	0.16	0.6172
dACC—>RSC	0.09	0.13	0.24	0.0109	RSC—>dACC	0.12	0.13	0.04	0.0632
dACC—>rDLPFC	0.11	0.12	0.22	0.0429	rDLPFC—>dACC	0.11	0.09	0.12	0.6773
MPC—>rPFC	0.14	0.21	0.32	0.0542	rPFC—>MPC	0.12	0.12	0.12	0.9995

*The p-values were given for the one-way ANOVA. The mean causality at each direction was also listed for each strategic group. The strongest causal influences among the three groups were marked in bold. The p-values of those with significant (p < 0.05/20, Bonferroni correction) group-difference were in bold*.

### Behavioral association of directional connectivity

**Figure 4:**
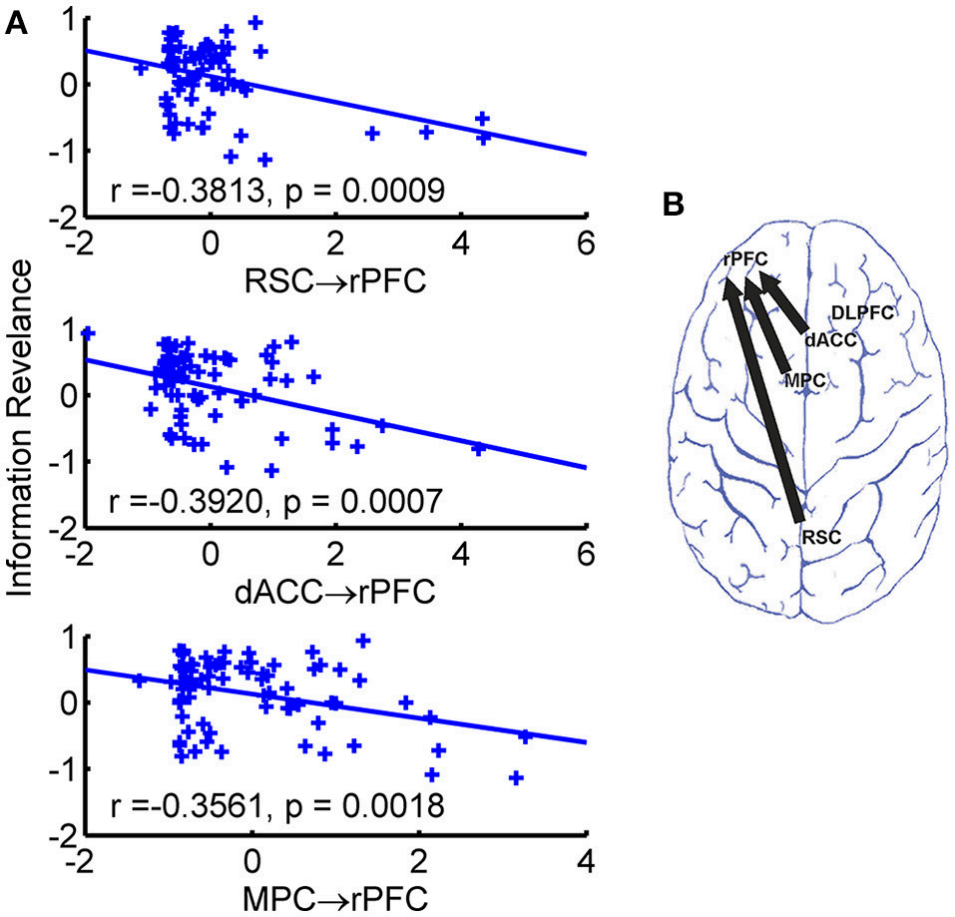
Significant behavioral associations of directional connectivity. **(A)** Scatter plot of directional connectivity against information revelation. The fitted least-square line was also shown. **(B)** Brain map of directional connectivity with significant behavioral association.

To investigate the behavioral correlation of the estimated directional connectivity, we calculated the Person's correlation coefficients between the strength of directional connectivity and the behavioral measurements (e.g., the information revelation and the R^2^). We found that after Bonferroni correction (*p* < 0.05/20) three directional connectivity of RSC → rPFC (*r* = −0.3813, *p* = 0.0009), dACC → rPFC (*r* = −0.3920, *p* = 0.0007), and MPC → rPFC (*r* = −0.3561, *p* = 0.0018), were negatively correlated with the slope, referred as buyers' information revelation coefficient (Figure 4), but not the fitness (i.e., the R^2^). The directional connectivity from rDLPFC to rPFC did not survive the Bonferroni correction, but the directional connectivity in this direction also negatively (*r* = −0.3485, *p* = 0.0029) associated with the information revelation. Moreover, neither IQ nor earnings of the game associated with the directional connectivity.

### Identification of social deception by neural signatures

**Figure 5:**
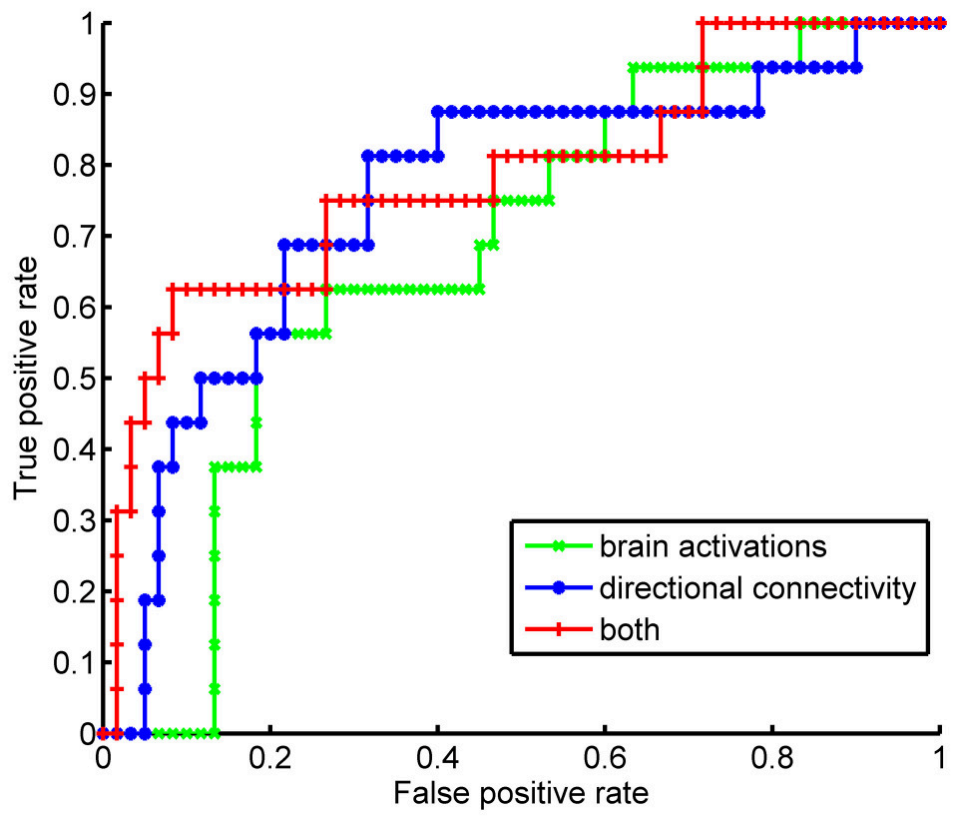
Performances of classifiers for strategic type by Receiver Operating Characteristic curve. The classifiers of strategist were trained by support vector machine with different sets of features, such as the estimation of brain activations at each ROI, or the estimated strength of directional connectivity among the selected ROIs, or both.

As both brain activations and directional connectivity were implicated in the bargaining strategy during the game, we tested if these brain signals can be used as a neural signature for the strategic deception. Consistent with our previous study (Bhatt et al., 2010), we also found the greater brain activations of rPFC (*p* = 0.0009 by ANOVA) and DLPFC (*p* = 0.0003 by ANOVA) during the game could be used as a neural signature of strategic deception. To assess the quality of these neural signatures (i.e., the brain activations and the directional connectivity), we trained SVM classifiers taking various neural signatures as input-features, including the activations of five brain regions, or the directional connectivity, or both. By a leave-one-out cross-validation procedure, we got 78.9% accuracy of identifying strategic deception by using the brain activations. Using the directional connectivity, we achieved 80.3% of classification accuracy. Combining both features of estimated levels of brain activations and strengths of directional connectivity, we improved the classification accuracy to 85.5%. The sensitivity and specificity of these classifiers were compared by the ROC curves (Figure 5). Features of brain activations gave an area under curve (AUC) as 0.6792, and directional connectivity achieved an AUC of 0.7573. Combined both features, we had an AUC as 0.7844. No significant difference was detected in AUC between these three classifiers. However, we confirmed the improvement in classification by adding the directional connectivity into the input-features of brain activations by both the (NRI, *p* = 0.0120) and the integrated discrimination improvement (IDI, *p* = 0.0007). These results suggest that directional connectivity lends extra neuronal support beyond brain activation to facilitate strategic deception during impression management.

## Discussion

Impression management has a significant impact on social interaction; different individuals employed distinct strategies to manipulate social images in others' mind. By a two-party bargaining game, we have exposed three types of strategies for impression management, but neural circuits supporting various social strategies remains unclear. Employing an advanced statistical model for causal inference, namely the Granger causality with SDN, we identified a significant pattern of directional connectivity among the key brain regions for social interaction during the game. We also identified the modulation of the directional connectivity by different social strategies. Especially, we showed that the strategists, who adopted a sophisticated, deceptive strategy to manipulate their social image, engaged stronger directional connectivity from both the RSC and dACC to rPFC during the game, and the connectivity strengths were associated with a behavioral indicator for the level of deception. To classify the strategic deception by neural signals, we found that the including the directional connectivity improved the classification accuracy of the classifier based on the brain activities. This significant improvement suggests that distinct patterns of connectivity among key brain regions underlying different social strategies.

These directional connections were used to identify individuals who adopted a strategic deception in the bargaining game. We have shown significant correlation between the strength of the directional connections (i.e., connections from RSC, dACC, and MPC to rPFC) and the buyers' information revelation (a behavioral index characterizing different strategies), suggesting that the sophisticated, deceptive strategy is supported by specific directional connectivity among key brain regions engaged in the impression management. Due to the limited number of subjects in each behavioral categories in the current sample, we could not use the k-fold cross-validation. Instead, we employed the leave-one-out procedure to assess the model performance, which is an unbiased estimation of the classification accuracy but with variance. Larger sample size is required in future experiments to further test this classification accuracy. Nevertheless, the current finding is along the same line as recent studies associating the directional connectivity with complex cognitive processes during social interactions. For example, moral processing on different issues invoked different directional connectivity (Cáceda et al., 2011), as justice issues (relative to care issues) were associated with stronger directional connectivity from both frontal pole (FP) and ACC to pSTS (posterior superior temporal sulcus), while care issue process was characterized by stronger connectivity from FP to ACC. Another recent example showed that motivation of empathy or reciprocity driving altruistic decision employed different directional connectivity (Hein et al., 2016). The empathy-motivated altruism was associated with a positive connectivity from ACC to anterior insular (AI) and reciprocity-based altruism was additionally related to positive connectivity from AI to both the ACC and ventral striatum. Notably, in both studies no brain activation was differentially modulated for different cognitive calculations, but the directional connectivity indicated distinct pathways of information flows among brain regions for the different calculations. Taken together, complex social cognition, such as impression management, or human motives, requires re-configuration of directional pathways for information flows in a brain network in addition to the magnitude of neural responses in the component brain regions.

Remarkably, the differences in directional connectivity identified by our use of the modified Granger causality (in consideration of SDN) capture the behavioral features exposed by the two-person bargaining game. During the game, the strategists adopt a forward-looking, longer-term strategy of manipulating their reputation in the eyes of their partners in order to increase their aggregate rewards–notably this strategy requires them to switch between “reputation-building” and collecting rewards depending upon the information in the current trial (Bhatt et al., 2010). When the value of the object is high, the strategists switch from building their reputation in their partner's mind to collecting rewards based on that reputation. However, it remains unclear what neural activity triggered this strategic switch. The stronger directional connectivity identified in the current study for the strategists suggested that the information flows from both the RSC and dACC to the rPFC were involved in the recalculation of more sophisticated strategy at rPFC (Knoch et al., 2006; Yoshida et al., 2010). This involvement was further supported by the observation that the strengths of the directional connectivity from both RSC and dACC to rPFC were positively associated with a behavioral indicator of social strategy during the game. The RSC has been related to prospective thinking (Vann et al., 2009), and the dACC has been thought to regulating cognitive control over goal-directed behavior (Bush et al., 2002; Brown and Braver, 2005; Carter and van Veen, 2007). Together, these results suggested that the stimulus driven attention (reflected by the directional connectivity dACC → rPFC, Bush et al., 2002) was combined with the prospective thinking (supported by the directional interaction RSC → rPFC; Vann et al., 2009) at the rPFC, and the repeated interactions between stimulus and response paved the way to a more sophisticated strategy. Alternatively, another possible explanation of the observed stronger directional influence of both RSC and dACC on the rPFC can be explained by an increased neural workload of the strategic deception, as the higher activation at the rPFC in the strategist group, instead of the higher engagement of the network. To demonstrate that the estimated directional connectivity did bring extra information for our understanding of the underlying neural basis of the strategic deception, we tested if including the directional connectivity could improve the classification accuracy for strategic deception. Indeed, we had observed a statistically significant improvement in the classification accuracy, which suggests that the directional connectivity brings extra information about the neural mechanism underlying the bargaining behavior.

The presence of the SDN in BOLD signal suggests an influence of one brain region on the variance in the brain activation of another region beyond the influence on its *mean* activation. As demonstrated in our previous paper (Luo et al., 2011), classical analysis of causality, which relied on the presumption of Gaussian white noise, failed to estimate reliable directional connectivity in such case. Several studies have discussed the potential importance of such SDN for the analysis of neural data both theoretically (Feng and Tuckwell, 2003; Tanaka et al., 2006; Kang et al., 2010) and experimentally (Harris and Wolpert, 1998; Jones et al., 2002; Luo et al., 2011). Our previous study showed an important role of the SDN in the modulation of taste circuit in the brain (Luo et al., 2013). The current study further confirmed that the presence of SDN in the fMRI signal, which was an important factor to be considered when developing methods to explore the neural circuitry of social interaction. The errors computed using the standard AR model showed significant correlation with the signal level in this data set, calling the results of classical Granger causality inferences into question. The sensitivity of these inference methods to this kind of noise is an important finding in itself (Luo et al., 2011).

GCA has been recognized as a promise method for fMRI based analysis of directional connectivity between brain regions, as long as its features are appropriately considered during interpretation of the results (Friston et al., 2013; Seth et al., 2015). The classical GCA on BOLD signal may have potential confounding factors, such as regional variation of HRF (David et al., 2008; Friston, 2009; Smith et al., 2011). However, it has been theoretically (Seth et al., 2013; Tao and Feng, 2016) and empirically (Schippers et al., 2011; Wen et al., 2013b) demonstrated that the results of GCA are reliable under moderate conditions, such as the neuronal delays between regions are above 1 s. Practically, cognitively meaningful associations of the directional connectivity estimated by GCA have been reported in task fMRI experiments (Wen et al., 2012, 2013a; Kadosh et al., 2016; Pu et al., 2016), and physiologically meaningful differences have also been observed in GCA-established directional connectivity (Hamilton et al., 2011; Palaniyappan et al., 2013; Ding et al., 2015). These previous successful applications of the GCA to fMRI experiment as well as the current work have a common nature that we focus on the changes in the Granger causality by considering it as a function of experimental settings, instead of trying to interpreting them at the hemodynamic level in neural terms. Nevertheless, these methods for causal inference are currently in their early stages and these results should be taken as suggestive. However, increasingly sophisticated methods for causal inference using fMRI data, such as the method applied in the paper, promise to significantly increase our understanding of neural function. The proposed method can be considered as one step ahead compared with the classic GCA. As pointed out in a most recent paper (Stokes and Purdon, 2017), the classic GCA does not dependent on receiver dynamics, which might be problematic for interpreting the cause and effect in neural systems. Different from the classic GCA, the proposed model assumes that the input noise process of the system is dependent on the signal of the system, and thereby both the cause and receiver dynamics are reflected in the resulting causality.

The value-dependent switch between reputation building and reward collecting is of particular interest in this game, but the current event-driven paradigm of the fMRI experiment only gave us limited data points for each condition (i.e., high or low value trial), especially when the decision time of the buyers was shortened (3 or 4 scans) at the second half of the game. Excluding the inter-trial data points, we had averagely 127 data points of each subject for connectivity analysis. With such a limited number of data points, we could not estimate reliable directional connectivity for the high and low value trials, respectively. Due to this reason, we also did not include the region right temporoparietal junction (rTPJ) in our model, as the activity of rTPJ was shown as value-modulated in our previous study (Bhatt et al., 2010). In addition, as the decision time differed from each subject, we could not align the trials across subjects, and thereby it is also difficult to investigate the dynamic changes of the directional connectivity during the game. These issues need to be addressed in the future studies.

In conclusion, different routes of the information flows support various brain functions and generate distinct behavior in response to environment. Here, we observed significant improvement in identification of strategic deception by including directional connectivity among key brain regions. The current work lends new evidence for the importance of directional connectivity in understanding sophisticated social cognition.

## Author contributions

QL and YM: Analysis and interpretation of data; drafting of manuscript. MB: Acquisition and analysis of data; drafting of manuscript. PM: Study conception and design; interpretation of data; drafting of manuscript. JF: Study conception and design; interpretation of data; critical revision.

### Conflict of interest statement

The authors declare that the research was conducted in the absence of any commercial or financial relationships that could be construed as a potential conflict of interest.
